# FEAR OF FALLING INTERACTS WITH ACTN3 GENOTYPE TO PREDICT FAST GAIT SPEED IN OLDER ADULTS

**DOI:** 10.1093/geroni/igae098.2808

**Published:** 2024-12-31

**Authors:** Allon Goldberg, Summer Burkhardt, Hayden Norris, Johnathon Garber, Natalie Little, Jaiden Kendall, Joseph Sucic

**Affiliations:** University of Michigan - Flint, Flint, Michigan, United States; University of Michigan - Flint, Flint, Michigan, United States; University of Michigan - Flint, Flint, Michigan, United States; Michigan State University East Lansing, East Lansing, Michigan, United States; University of Michigan - Flint, Flint, Michigan, United States; University of Michigan - Flint, Flint, Michigan, United States; University of Michigan - Flint, Flint, Michigan, United States

## Abstract

Deficits in walking speed are associated with poor health outcomes in older adults. The alpha-actinin-3 gene (ACTN3) harbors the R577X polymorphism, which influences muscle function and physical performance in older adults. Fear of falling (FOF) is associated with gait, mobility and physical performance deficits in older adults. Our objective was to determine if effects of FOF on fast gait speed (FGS) are associated with ACTN3 genotype, to identify individuals based on ACTN3 genotype at risk for greatest declines in FGS with onset of FOF. Older adults (N=168, female=61.3%, mean age 72.8 years, range 65-85 years) attended a study visit at which demographic, self-rated health (SRH) and FOF information was collected, and 10-meter FGS was tested. Saliva was obtained for ACTN3 genotyping. Multiple regression evaluated variance in FGS explained by FOF, RR genotype (present/absent), and interaction of FOF with RR genotype, controlling for age, sex and SRH. The final regression model explained 36.9% of FGS variance (p<.001). FOF was a significant predictor (β=-.163; p=.026) of FGS. RR genotype did not independently predict FGS (β=.103; p=.161), but was interactively with FOF a significant predictor (β=-.181; p=.024) of FGS. The effect of FOF on FGS was greater in individuals with RR genotype than in the RX/XX genotype subgroup (partial eta squared 0.083 and 0.030 respectively). The significant FOF x RR genotype interaction suggests that effects of FOF on FGS vary based on ACTN3 genotype. Individuals with RR genotype (compared to the RX/XX subgroup) may experience greater FGS declines with onset of FOF.

